# Identification and Management of COVID-19 Related Child and Adolescent Mental Health Problems: A Multi-Tier Intervention Model

**DOI:** 10.3389/fpubh.2020.590002

**Published:** 2021-02-04

**Authors:** Gunjan Dhonju, Arun Raj Kunwar, Utkarsh Karki, Narmada Devkota, Isha Bista, Rampukar Sah

**Affiliations:** Child and Adolescent Psychiatry Unit, Kanti Children's Hospital, Kathmandu, Nepal

**Keywords:** COVID-19, capacity building, system level intervention, child and adolescent mental health, teleconsultation

## Abstract

Nepal is a low and medium-income country (LMIC), situated in South-east Asia, with a population of 29 million, of which, 40–50% are children and adolescents. The Coronavirus Disease 2019 (COVID-19) pandemic has affected the lives of people around the world, including Nepal. The child and adolescent mental health (CAMH) needs and services in Nepal have a significant gap. CAMH in Nepal suffers from lack of specialized training in this field as well as scarcity of human resources and services. There is only one full-time child and adolescent psychiatry (CAP) out-patient clinic in the country. Some recent activities have focused on CAMH in Nepal but the COVID-19 pandemic has produced new challenges. Access to mental health services for children and adolescents (C&A) across Nepal has been adversely affected. Factors such as closure of schools, confinement at home, lockdown, transportation problems, uncertainty, loss of usual routine and fear of infection have affected the mental health of C&A. This has highlighted a need to build capacity of available local human resources, enhance community support, teach measures of coping with stress and improve CAMH service delivery by strengthening the referral system, but these have to be addressed overcoming problems of travel restrictions and limited resources. To address these needs, online platform can be a suitable approach. With this view, a multi-tier CAMH intervention model was developed, which utilizes online platform for training mental health professionals across Nepal, who would then facilitate sessions for C&A, teachers, parents and caregivers; and link them to CAMH services locally, and remotely through teleconsultation. This started as a pilot from June 2020 and will continue till end of February 2021, with the aim to reach 40,000 C&A, parents, teachers and caregivers. As of Nov 2020, this model has been used to successfully conduct 1,415 sessions, with 28,597 population reached. Among them, 16,571 are C&A and 12,026 are parents, teachers and caregivers, across all 7 provinces of Nepal. In this paper, the multi-tier intervention to address the COVID-19 related CAMH problems has been discussed as a feasible framework for resource limited settings and LMICs like Nepal.

## Introduction

Nepal is one of the low and middle-income countries (LMICs), situated in South-east Asia, between India and China, with a population of around 29 million. Children and adolescents (C&A) make up 40–50% of Nepal's population, 87% of which live in rural settings (1). With <5% of the national budget spent on health, and only 1% of the national health budget allocated to mental health, investment in child and adolescent mental health (CAMH) remains scarce in Nepal (2). The country's C&A health programme largely focuses on management of diarrhoeal diseases, malnutrition, malaria, respiratory illness, and adolescent reproductive health. There is only one full-time child and adolescent psychiatry (CAP) service provider available in the country, as part of the out-patient department (OPD) of Kanti Children's Hospital (KCH), situated in the capital city Kathmandu, established in 2015 after the major earthquake in Nepal (3). This CAP OPD provides CAMH services to C&A from all parts of Nepal and has been the focal point of CAMH services for Nepal.

In December 2019, the Ministry of Health and Population (MoHP) endorsed and disseminated the “Child and Adolescent Mental Health Training Manual for Doctors, Nurses and Paramedical Professionals,” as module 3 of community mental health care package (4). This was developed in collaboration with the CAP team at KCH and United Nations Children's Fund (UNICEF), under the leadership of MoHP, Government of Nepal. This CAMH module is a 5-day on-site training of doctors and paramedical professionals across Nepal. The roll-out of this training aims to build capacity of medical and paramedical professionals to improve task-shifting, early identification, local intervention and referral of CAMH problems. However, implementation of this CAMH training was put on hold due to the COVID-19 pandemic.

A study on early career psychiatrists' (ECPs) perspective in child and adolescent psychiatry training in Nepal showed that 44% reported CAP training exposure as inadequate and 74% desired for additional training (5). Additionally, they reported that on a 10-point scale, their confidence in management of CAP cases was 4.58 ± 1.59.

Nationwide lockdown due to COVID-19 pandemic began in Nepal during March 2020, with restriction of both air and road travels, as well as closure of borders with India and China (6). All people including C&A were confined to their homes, and schools were closed. The government lifted the lockdown and businesses opened, but schools still remained physically closed as of November, 2020. Some schools, mostly those privately run, resumed classes through online platforms while schooling remains disrupted for the majority of students in Nepal. In Nepal, deficient funding for mental health services, increased social media use, suddenly imposed lockdown, poor understanding of lockdown restrictions, sudden student-life changes and postponement of exams were stated as risks for COVID-19 related mental health problem for the young population (7).

The COVID-19 pandemic has taken a significant toll on CAMH and access to mental health services have been negatively impacted (8, 9). Based on UNICEF U-Report Poll data collected among adolescents and youth in Nepal during July of 2020, 43% of respondents reported feeling stressed and 19% sad. Additionally, 74% respondents felt fearful that their family members or themselves could become infected with COVID-19. Furthermore, 80% felt that studying during this period was challenging, with 28% being most worried about exams and 18% finding it difficult to study in their home environment (10).

The impact of COVID-19 on CAMH and restricted access to available support systems and services highlight a critical need to reach C&A as well as their caregivers across Nepal. The CAP team of Kanti Children's Hospital responded by developing and piloting a multi-tiered CAMH intervention in partnership with UNICEF Nepal. This paper outlines the key components of the multi-tiered CAMH intervention model and experiences with its implementation till date during the COVID-19 pandemic.

## Overview of the Multi-Tiered CAMH Intervention Model

In this intervention model, COVID-19 has been taken as one of the stressors that could adversely affect CAMH. The model incorporates basic psychosocial support for management of stress, tailored more toward COVID-19 related stress, but is not limited to it. This also includes identification and management of CAMH problems locally, and remotely through link with tele-consultation services. The same framework can be used for management of CAMH issues due to other stressors as well. This is a multi-tier model because it includes training of mental health professionals by master trainers through Training of Trainers (TOTs) sessions, and the TOT recipients will then conduct sessions with C&A, parents, teachers, care givers.

Although the primary target population are C&A who, through these sessions, are empowered to manage their own stress, this model also ensures CAMH awareness and support at levels of teachers, parents, caregivers. Then, there is possibility of a consultation with the mental health professional/ facilitator when needed, as they would have been linked by the session, this may encourage help seeking behavior. This is further linked to the higher center for specialized CAMH services via tele-consultations. Thus, the C&A will be able to manage their mental health at 4 different tiers. The 4 tiers of the intervention model have been summarized in Table 1 below.

**Table 1:** Multi-tiered CAMH Intervention.

**Tiers**	**Description**
One	Children and adolescents in different parts of Nepal
Two	Parents, Teachers, and Caregivers in different parts of Nepal.
Three	Mental health professionals: psychiatrists and psychologists working in different parts of Nepal. They have an MD degree in Psychiatry or Master's degree in Psychology. Their work includes mental health services primarily targeting adults.
Four	Child and Adolescent Psychiatry team at Kanti Children's Hospital. This comprises a team of child and adolescent psychiatrists, and clinical psychologists. They have post-MD degrees of specialization in Child and Adolescent Psychiatry and Post-master's degrees of specialization in clinical psychology, respectively. This team exclusively works in the field of CAMH.

**Figure 1:**
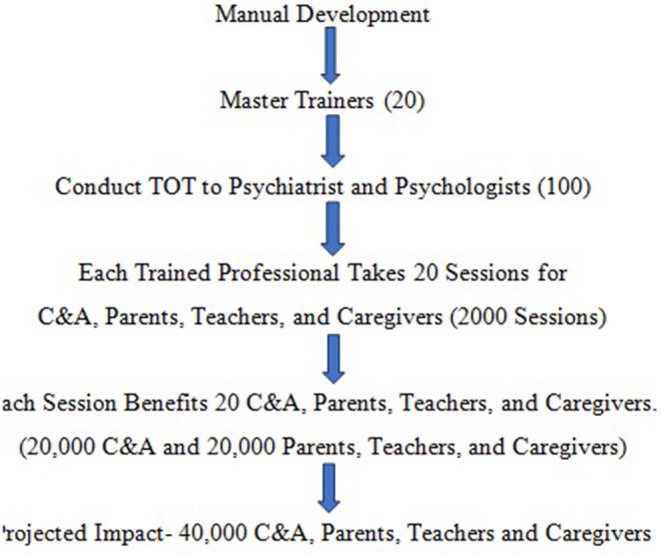
Flowchart of multi-tiered CAMH intervention phases.

The 3 phases the COVID-19 CAMH Intervention pilot include: (1) *Manual Development*, (2) *Training*, and (3) *Outreach to C&A, Caregivers, and Teachers*, as illustrated in Figure 1 below. The pilot aims to train 20 master trainers, 100 Psychiatrist and Psychologist who would facilitate sessions to reach a total of 40,000 C&A, Caregivers and Teachers from June, 2020 to February, 2021.

## Phase 1: Development of a Manual on COVID-19 Related CAMH Problems Identification and Management

Considering the limitations in the context of the COVID-19 pandemic, yet immediate demands, there was need for a simple but impactful intervention model that could be implemented over a short period of time and reach different parts of the country. There was also the need to train mental health professionals on CAMH. This led to development of a COVID-19 related CAMH Training Manual in a Training of Trainer's (TOT) format. This has two modules: (a) Module for Session with Teachers, Parents, and Caregivers; (b) Module for Session with Children and Adolescents.

After the development of the initial draft of the manual, trail sessions were conducted. One trial session, via online platform through Zoom application, was done for 10 teachers from various schools at Kailali District, which is a remote district in Nepal. Online trial sessions were also conducted in a school in Kathmandu, the capital city of Nepal, with 80 teachers and 600 students, including parents for younger children. Similarly, other online trial sessions were conducted for C&A and staff in child-care homes and Child Helplines in the capital city. Based on our observations, and feedback received from participants (including C&A), the Training of Trainer's manual was updated. This second draft of the manual was used to conduct online trial TOT sessions for mental health professionals comprising of 19 psychiatrists and psychologists. Then, the manual was further updated and finalized based on the inputs from these professionals. The session content of the COVID-19 related CAMH training manual is summarized in Table 2.

**Table 2:** COVID-19 CAMH training manual content.

**SN**	**Heading**	**Content**
i	Introduction	The facilitator introduces oneself to the participants and the participants introduce themselves.
ii	Privacy and Confidentiality	No participant is forced to speak, if they wish to not share anything, this is respected. Also, it is mentioned that any personal information shared within the session, will remain within the session and not shared to others. If anyone wishes to contact the facilitator to discuss some issues in private, this opportunity is given by providing contact details at the end of the session.
iii	Ventilation	This session allows the participants to express their thoughts, feelings and worries related to the COVID-19 pandemic.
iv	Clarification of COVID-19 related queries	These are done through discussions, stories, or informational videos.
v	Discussion of COVID-19 Pandemic as a Stressor	Discussions on how COVID-19 pandemic is affecting mental health of C&A, teachers, parents and caregivers.
vi	First Level Response (11)	These refer to the first verbal responses provided to the C&A, parents, teachers or caregivers for the concerns they express. This follows the steps of *Acknowledgment, Universalization, Validation and Empathy*. These headings are discussed in brief to provide a clear concept. Role plays are done with volunteers from the participants, and also demonstrations by facilitator, to practice first level response to the problems expressed by C&A or those perceived by teachers, parents and caregivers.
vii	Identification of Symptoms of Stress Related to COVID-19	These are discussed in three general categories as emotional symptoms, behavioral symptoms and physical symptoms.
viii	CAMH Intervention Phases	These include discussions on maintaining a structured routine, proper diet, regular exercise, learning activities, indoor games, creative and fun activities. Besides these, relaxation exercises and breathing exercises are practiced in the session.
ix	Identification of Need to Seek Help, Promotion of Help-seeking Behavior and Referral	This focuses on identifying socio-occupational dysfunction, and any high-risk situations such as self-harm, suicidal tendencies or risk of harm to others. If identified or suspected, then referral to a mental health practitioner or medical facility is advised.
x	Link with Tele-consultation Services, Supervised by CAP team at Kanti Children's Hospital	In case, there is a need for consultation with specialized child and adolescent psychiatry team, these are arranged via zoom platforms. The CAP team can also provide assistance to the local mental health professionals for management of such cases through tele-consultation. The session provides the participants with contact numbers for local mental health services, as well as child helpline number, other mental health hotline numbers, and CAP OPD hotline numbers.
xi	Materials and Methods	Videos on COVID-19, presentation slides, stories, workbooks, role plays, and discussions are used during the sessions to make the sessions interesting. These materials are also provided to participants via mails or downloadable links.

## Phase 2: Training

### Master Trainers

The master trainers consist of Child and Adolescent Psychiatrists, and Clinical psychologists from CAP team at KCH, and some mental health professionals involved in development of the manual. The pool of master trainers can later be increased by including some of the TOT recipients after observation of their sessions.

### Training of Trainers (TOT)

The TOT sessions are provided by master trainers to mental health professionals such as psychiatrists and psychologists from different parts of Nepal.

The announcements for TOTs were made on social media platforms like Facebook groups associated with the mental health professionals and via email correspondence. These mental health professionals were recruited for TOTs through online registrations via applications like Google Forms. After registration, email correspondences were made about details of TOT session schedules.

The TOTs are conducted online, through tele-video platforms such as Zoom application, with participants from different parts of country. Each module of the manual is discussed over a period of 3 h; the TOT takes 2 days with a total of 6 h.

## Phase 3: Outreach to C&A, Teachers, Parents, and Caregivers

Parents are reached out through schools or through community organizations. Teachers and students are reached out through school administrations. Caregivers here refer to staff of child care institutions and organizations, such as- orphanages, child-care homes, child helplines, etc., who work with children, The Caregivers are reached out through respective organizations.

Each of the 100 TOT recipients can conduct sessions for teachers, parents and caregivers on COVID-19 related CAMH issues identification and management through one and half hour sessions. Each of the 100 TOT recipients can provide sessions for C&A on identifying and managing COVID-19 related CAMH issues through one and half hour sessions. Tele-supervision and monitoring of these sessions by master trainers ensure quality of sessions by the TOT recipients, and constructive feedbacks are given to facilitators. After few sessions, they conduct session independently.

Each TOT recipient is expected to conduct around 20 sessions, although some may take more and some less. The target is to conduct 2,000 sessions, with around 20 participants in each session. This would amount to 40,000 total population reached, with roughly 20,000 C&A, and 20,000 teachers, parents and caregivers at the end of the pilot in February, 2021.

## Delivery Platform

Given COVID-19 social distancing requirements, this intervention model uses the online platform, while also allowing for in person delivery. Online sessions were used for conducting training of trainers (TOT) sessions for mental health professionals, as well as sessions for C&A, teachers, parents or caregiver groups. Various applications such as Zoom, Microsoft Teams, Google Hangouts were utilized. Some of these are also available in free versions, so do not have financial implications.

Sessions could also be conducted in physical setting with appropriate COVID-19 related safety measures in place. This is a necessity in settings where online platforms are not feasible due to internet or device unavailability. This can also be continued in schools as sessions for C&A and teachers, once schools resume. The school platform is an important entry point for providing support to C&A with COVID-19 related stress and for assistance in adjustment with school re-entry. Similarly, sessions can be conducted in community spaces for parent groups, youth clubs, mothers' groups, childcare institutions and other community-based organizations.

## Incentives

Incentives are provided to the TOT recipients for participating in the TOTs, these are in the form of mobile-phone top-ups of NRS 500 (Nepalese Currency), equivalent to 4.23 US dollars, to cover the internet cost for attend the sessions. Incentives are also provided to TOT recipients for facilitating sessions with C&A, teachers, parents and caregivers. Financial incentive of NRS 1,500, equivalent to 12.70 US dollars, was provided to the TOT recipients for each session facilitated by them. It is hoped that this would have sensitized them for sustainable continuation of such sessions in future by collaboration at local levels.

## Tele-Consultation Workspace

Tele-consultations with C&A patients are currently being conducted through the CAP OPD at KCH or home settings. A separate workspace is being established which will have the required equipment to ensure smooth running of the sessions and a back-up of the data will be set up to ensure safe storage. This will help to reduce the technical problems of occasional internet connectivity problems or power cuts.

## Reach and Coverage

Since the project commencement in June, 2020 there has been encouraging progress in reaching the targeted population. TOT sessions have been completed for 100 mental health professionals. At the end of November, 2020, within a period of 5 months, the TOT recipients have conducted 1,415 sessions, with 28,597 population reached. Among them, 16,571 are C&A, and 12,026 are parents, teachers and caregivers. With this pattern, it seems possible to reach 40,000 in the next 3 months by end of February, 2021. The project has successfully reached C&A, teachers and parents across all 7 provinces in Nepal, although there are variabilities in distribution of sessions due to availability of internet connection and remoteness of some places.

## Discussion

The COVID-19 CAMH intervention model provides a suitable framework for the current scenario of the COVID-19 pandemic, where physical distancing has to be maintained, and groups have to be avoided. It overcomes the risk of contracting infection with COVID-19, while still providing mental health support. This model can be useful to generate awareness on mental health, identify mental health problems, teach measures to manage the problems locally, and become aware of available mental health services. This creates a local network of supporting elements to safeguard mental health of children and adolescents. This also builds the capacity of the existing mental health professionals and enhances their collaboration on promoting CAMH. This type of project can be conducted in low resource settings like Nepal. It helps to overcome issues of scarce manpower and lack of funds. Being a short training, both for TOT sessions as well as for sessions for C&A, teachers and parents, there is less risk of discontinuation of participation. The session content and flow has been made simple, for ease of conduct as well as ease of understanding for the participants; yet, it can have an important role on safeguarding mental health of children and adolescents as well as that of parents, teachers and caregivers.

The COVID-19 CAMH intervention model developed out of desperate necessity. Although the current numbers reached are only a fraction of the total C&A population of Nepal, this has presented as a useful model that can be scaled up to reach more C&A nationwide. Collaboration with local NGOs, community organizations and mental health fraternities have been extremely important for the project, and have made it progress with more ease than anticipated. However, hiring of personnel for logistics is necessary to handle the paperwork, keep records of sessions, provide renumerations and other managerial aspects of the project.

Reaching out to the mental health professionals was initially difficult; however, with time the number of required professionals were met. It is difficult to reach the population of remote places where internet connection is poor, and the availability of smart-devices is also variable. Minor issues with internet connectivity, break in voice and lag time to buffer have been experienced occasionally. Arranging a convenient time for all participants might be difficult, so number of participants in sessions may vary from those who have registered. Some of the participants were using zoom for the first time, so unfamiliarity with the online setting and application use may be an issue initially. The online experience may be different from in-person training. There are less opportunities for the participants to interact with each other besides during the training.

While many schools in the urban areas have started online platforms, these can be utilized for conducting the sessions, but online platforms are still not available in majority of the schools in the rural settings and also some schools in urban settings. So, for many, a physical setting has to be utilized after the school resumes.

Sometimes, these training sessions may present as extra burden on parents, teachers and caregivers due to their already busy schedules. Lack of awareness and stigma related to mental health problems, and disinterest in associating with mental health professionals may also be present.

## Need for Evaluation of the Intervention Model

Although the COVID-19 intervention model has been applied to reach the target population, it is important to evaluate the impact of the model and intervention provided to understand its effectivity in the long run. In order to achieve this, after the end of the project at February, 2021, an evaluation will be undertaken.

## Conclusion

Incorporation of tele-consultation services and remote CAMH services can help to bridge the gap in needs and services, thus addressing the problems with availability, accessibility, as well as affordability of these CAMH services. With cooperation from schools, community, local authorities and coordination with other partner organizations working in mental health, this could be implemented in a larger scale.

If the evaluation of the intervention model shows good effectiveness, this model can be considered for a scale-up to be used in all schools in Nepal by coordinating with the health and education sectors at government levels. This can be continued even after the COVID-19 pandemic subsides, such as in situations of natural calamities like earthquake, landslides, floods as well as stressful situations in home, school and community; thus, promoting the overall child and adolescent mental health.

## Data Availability Statement

The raw data supporting the conclusions of this article will be made available by the authors, without undue reservation.

## Ethics Statement

Since the intervention model described is a programme implementation model and not a study or a research, ethical approval from an Institutional Review Board (IRB) was not needed. However, practices of ethical conduct are advised to be followed by all mental health professionals facilitating the sessions, along with others in the team.

## Author Contributions

GD and AK contributed in conceptualization and design of the COVID-19 CAMH intervention model, development of the training manual, drafting and editing of this manuscript. UK contributed in development of the training manual, drafting and editing this manuscript. IB, ND, and RS contributed in development of the training manual discussed in the manuscript.

## Conflict of Interest

The authors declare that the research was conducted in the absence of any commercial or financial relationships that could be construed as a potential conflict of interest.
